# Vertical Distribution of Butterfly Community (Lepidoptera) and Its Drivers on Mount Gongga, Western China

**DOI:** 10.3390/insects17090890

**Published:** 2026-08-25

**Authors:** Zhuoyuan Wang, Shanyong He, Lei Bai, Zhaolong Wang, Jie Zhang, Xiushan Li

**Affiliations:** 1 Key Laboratory of Wildlife Conservation in Southwest China of the Ministry of Education, College of Life Sciences, China West Normal University, Nanchong 637009, China; zhuoyuan0620@163.com (Z.W.);; 2 The Station of Qingyang Forest Pest Control and Quarantine, Qingyang 745099, China; 3 Department of Animal Ecology and Tropical Biology, Julius-Maximilians-University of Würzburg, Am Hubland, 97074 Würzburg, Germany; jie.zhang@uni-wuerzburg.de

**Keywords:** butterflies, elevational distribution pattern, driving factors, Mount Gongga, biodiversity conservation

## Abstract

Butterfly elevational distribution patterns are modulated by temperature stratification, vegetation composition, butterfly functional traits, and anthropogenic disturbances. To explore the elevational patterns of butterfly α- and β-diversity and their driving mechanisms on Mount Gongga, we established 18 standardized 1000 m transects at 200 m intervals across an elevational range of 1000–4500 m. Butterfly species richness and abundance were systematically surveyed using the line-transect method. The results indicated that both butterfly species richness and abundance significantly decreased with increasing elevation. Notably, butterfly β-diversity exhibited a distinctive nonlinear trimodal pattern along the elevational gradient, with three peak values occurring at 1900–2300 m, 3500–3900 m, and 4300–4500 m. This pattern differs substantially from the monotonically decreasing or unimodal β-diversity trends widely reported for most mountain insect communities. Vegetation ecotone effects, habitat heterogeneity differentiation, elevational species turnover, and topographic microclimate heterogeneity collectively drive this unique distribution pattern. Based on these findings, we propose targeted conservation recommendations: (1) prioritize the protection of key vegetation transition zones; (2) sustain vegetation integrity to guarantee sufficient food and habitat resources for butterflies; (3) address climate change threats to conserve high-elevation endemic butterfly species; (4) develop long-term monitoring programs to improve regional biodiversity conservation systems.

## Simple Summary

Exploring the elevational patterns of species alpha and beta diversity is essential for disentangling how environmental filtering and human disturbance mediate ecological community assembly, thereby supporting the formulation of targeted biodiversity conservation strategies. We aimed to explore the distribution pattern of the butterfly community on Mount Gongga. The results shown that with increasing elevation on Mount Gongga butterfly α-diversity significantly decreased. While β-diversity exhibited a nonlinear trimodal pattern. Vegetation ecotone effects, habitat heterogeneity differentiation, high elevational species turnover, and topographic microclimate heterogeneity collectively drive this unique distribution pattern. Conservation recommendations: prioritize the protection of key vegetation transition zones; To address climate change threats to conserve high-elevation endemic butterfly species; To develop a long-term monitoring program to improve regional biodiversity conservation systems.

## 1. Introduction

The distribution patterns of biodiversity are jointly regulated by the interactive effects of climate change, vegetation distribution, and anthropogenic disturbances [1,2,3,4,5]. As a fundamental geographic factor structuring mountain biodiversity, elevation acts as a strong environmental filter that sorts species abundances and shapes distinct biological assemblages across vertical vegetation zones [5]. Along elevational gradients, gradual declines in ambient temperature drive systematic vertical succession of mountain vegetation, ranging from farmland and evergreen broad-leaved forests at low elevations to mixed coniferous–broad-leaved forests, coniferous forests, alpine meadows and alpine scree sparse vegetation belts at high elevations [6] Accordingly, the richness and composition of animals and plant species inhabiting this zone will change correspondingly. Correspondingly, human disturbance regimes also vary vertically: agricultural cultivation dominates low-elevation landscapes and alters local biodiversity [7], while livestock grazing represents the primary anthropogenic pressure on high-elevation shrublands and alpine grasslands [8]. The stratified variation in environmental conditions and human activities collectively shapes the unique elevational distribution patterns of mountain species.

Elevational species richness follows four main patterns—monotonically decreasing, low-altitude plateau, low plateau with a mid-elevational peak, and mid-elevational peaks [5,9]. Such heterogeneous distribution patterns are predominantly governed by elevational variation in temperature and sunshine duration, the diversity and abundance of host and nectar plants, and species-specific functional traits [6,10]. In this context, exploring the elevational patterns of species alpha and beta diversity is critical for disentangling how environmental filtering and human disturbance mediate community assembly, which further supports the development of targeted biodiversity conservation strategies.

Climate change is one of the most pervasive drivers altering mountain plant and animal communities worldwide [11,12]. Ongoing climatic warming substantially reshapes species spatial distributions and modulates the rate of global biodiversity loss. In the Northern Hemisphere, numerous taxa have exhibited consistent range shifts toward higher latitudes and higher elevations, leading to elevated species richness at mountain summits and high-latitude regions [13,14,15]. Beyond simple range shifts, elevational variation comprehensively regulates multiple ecological processes, including species distribution patterns, ecosystem structure and function, organismal adaptation and life-history strategies, and community stability [16,17]. Elucidating these elevational effects is essential for understanding the mechanisms underlying biodiversity formation and maintenance, and provides empirical support for regional biodiversity conservation and sustainable management.

Vegetation composition constitutes another core determinant of mountain biodiversity patterning. In high-relief mountain systems, vegetation exhibits prominent vertical zonal differentiation, with discrete vegetation belts forming continuous gradient sequences from low to high elevation [6]. Each vegetation belt differs significantly in species composition, community structure, and ecological function, providing heterogeneous habitats, food resources, and microenvironmental conditions for wild fauna [6]. Such vertical vegetation stratification further differentiates animal community composition and species turnover across elevations, ultimately structuring the vertical biodiversity patterns of mountain ecosystems. A positive influence of forest cover on butterfly species richness and abundance in the surroundings was found in many studies [12,17,18,19,20,21,22,23].

Anthropogenic disturbance exacerbates biodiversity decline across elevational gradients with distinct mechanisms at different elevation zones. Low-elevation agricultural activities simplify vegetation structure and reduce plant community complexity, resulting in impoverished faunal biodiversity [7]. In contrast, high-elevation alpine meadows suffer severely from overgrazing, which degrades natural habitats, weakens ecosystem service capacity, and induces significant losses in species richness and abundance [8].

Butterflies (Lepidoptera) are highly sensitive bio-indicators of climate fluctuation and habitat degradation, and their diversity and community composition effectively reflect ecosystem responses to external perturbation [6,24]. Mountain butterfly assemblages are regulated by divergent drivers along elevation: lowland communities are predominantly constrained by anthropogenic activities, while high-altitude assemblages are primarily limited by harsh climatic conditions. Mountains serve as critical refugia for butterfly diversity, hosting 3.5 times more butterfly diversity hotspots than lowland regions and supporting a high proportion of montane-endemic species [4].

Climate change profoundly modulates multiple biological dimensions of butterflies, including phenological rhythms, elevational distribution ranges, and key functional traits such as body coloration, wing morphology, thermal regulation capacity, and reproductive performance [25,26,27,28,29,30]. Consistent with general mountain biota, Northern Hemisphere butterflies shift their ranges upward and poleward under climate warming. Nevertheless, dispersal barriers and severe high-altitude environmental filtering limit population colonization and persistence, causing continuous population regression and increased extinction risk for most butterfly species [4,31]. Consequently, butterfly alpha diversity generally declines with increasing elevation across mountain systems [29,32,33,34].

Plant resource availability strongly governs butterfly diversity and community assembly across elevations, affecting both larval and adult life stages [35,36,37,38]. Adult butterflies rely on nectar plant resources for survival and reproduction, whereas specialized caterpillars depend entirely on host plant availability, making herbaceous plant richness a key indicator of habitat suitability for butterfly populations [39]. Together with vegetation heterogeneity, elevation acts as a fundamental modulator of butterfly diversity, community turnover, and plant–butterfly interaction networks in mountain landscapes.

Although elevational patterns of butterfly diversity and their driving mechanisms have been well documented across European and American mountain systems, relevant empirical evidence remains severely limited in the mountain regions of western China. To fill this research gap, the present study investigated butterfly diversity patterns along a comprehensive elevational gradient on Mount Gongga, a globally recognized biodiversity hotspot in southwestern China. By clarifying butterfly elevational distribution patterns, community assembly processes, and responses to environmental variation, this study improves our mechanistic understanding of mountain butterfly persistence under ongoing climate change and provides practical implications for regional insect biodiversity conservation. We formulated three core hypotheses: (1) Butterfly alpha diversity declines monotonically with increasing elevation, while beta diversity peaks at mid-elevations. (2) Elevationally stratified vegetation belts support distinct host plant assemblages, driving strong butterfly community differentiation and high species turnover across elevational zones. (3) Elevation is the primary driver of butterfly community composition, filtering species into discrete low- and high-elevation assemblages with limited cross-elevational distribution.

## 2. Material and Methods

### 2.1. Study Area

**Figure 1:**
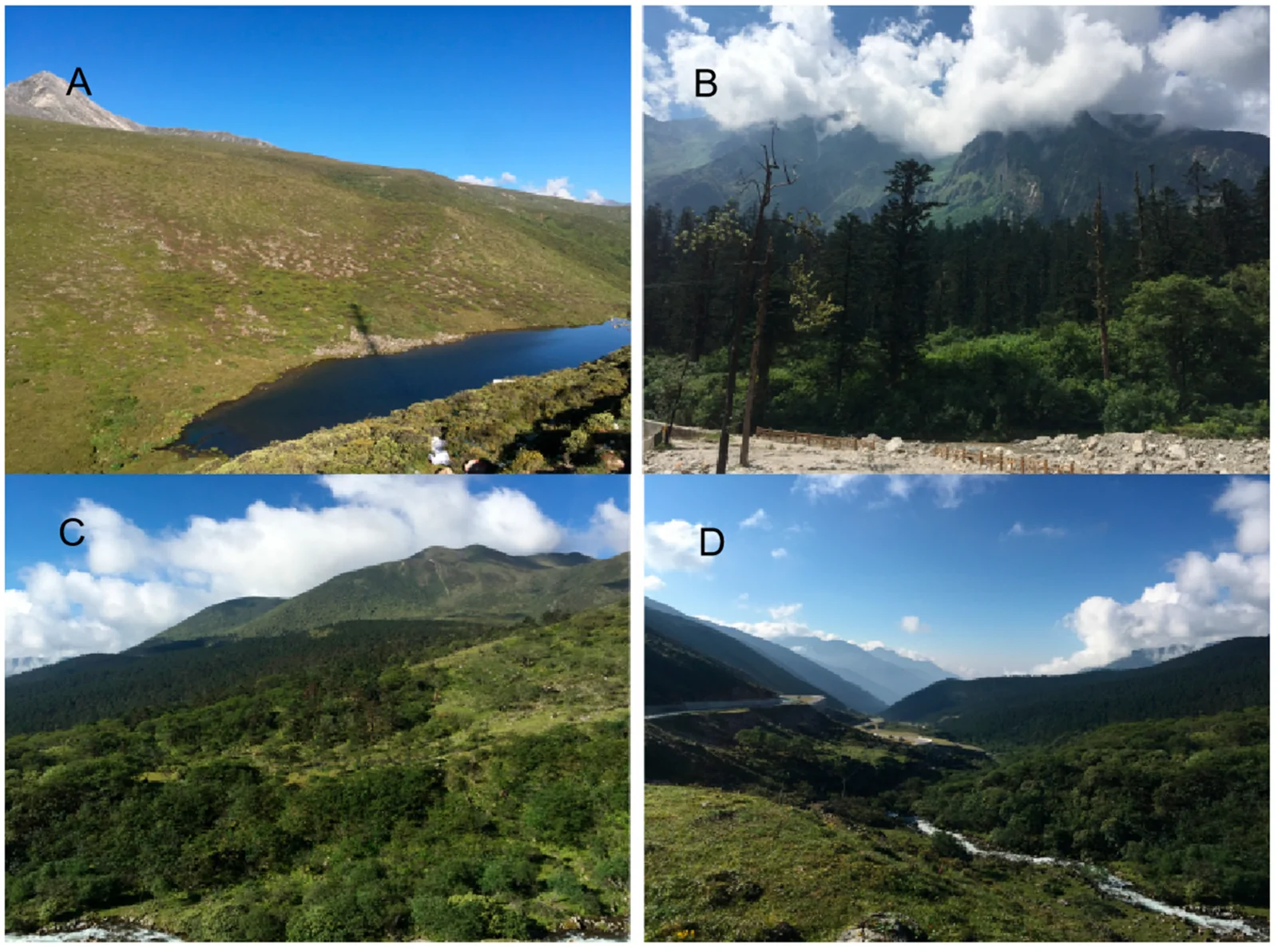
Vertical distribution of vegetation zones. (**A**): Alpine meadow; (**B**): dark coniferous forest, (**C**): shrubland, (**D**): mixed evergreen–deciduous broadleaf forest.

This study was carried out on the eastern slope of Mount Gongga (Figure 1), a high-elevation mountainous region in Luding County, Sichuan Province, western China. Located in the central Hengduan Mountains, as the core area of the Himalaya–Hengduan Mountains, Mount Gongga harbors abundant biological resources and represents an ideal research area for exploring the origin and evolution of wildlife. Featuring complex terrain and a humid climate, this region supports extremely rich biodiversity and is listed as one of the 36 global biodiversity hotspots identified by Conservation International (CI: https://www.conservation.org/learning/biodiversity-hotspots) (URL accessed on 20 August 2026). Referred to as the “King of Sichuan Mountains”, Mount Gongga is the highest peak of the Hengduan Mountains on the eastern Qinghai–Tibet Plateau. Standing at 7556 m, its main summit is counted among the world’s renowned high peaks [6,40,41].

The eastern slope of Mount Gongga possesses an elevational drop of approximately 6400 m, featuring a typical alpine canyon landform. Influenced by the Pacific monsoon, the region has a warm and humid climate with distinct vertical vegetation zonation. Our research area includes five vegetation belts: an evergreen broad-leaved forest (EBF) zone at 1100–1700 m a.s.l. dominated by *Cinnamomum*, *Phoebe*, *Lithocarpus* and *Cyclobalanopsis*; a mixed evergreen–deciduous broadleaf forest (MEDBF) at 1700–2500 m a.s.l. composed of *Tsuga*, *Betula*, *Acer* and other tree species; a coniferous and broadleaf mixed forest (CBF) at 2500–2900 m a.s.l. mainly consisting of fir (*Abies*), spruce (*Picea*) and *Betula*; a coniferous forest (CF) zone at 2900–3700 m a.s.l. mainly consisting of fir (*Abies*) and spruce (*Picea*) forests; and an alpine shrubland and meadow (SM) zone at 3700–4500 m a.s.l. comprising alpine shrubs such as *Rhododendron*, *Salix* and *Juniperus*, as well as alpine meadow species including *Kobresia*, *Festuca* and *Allium* [6].

### 2.2. Transect Counting

**Figure 2:**
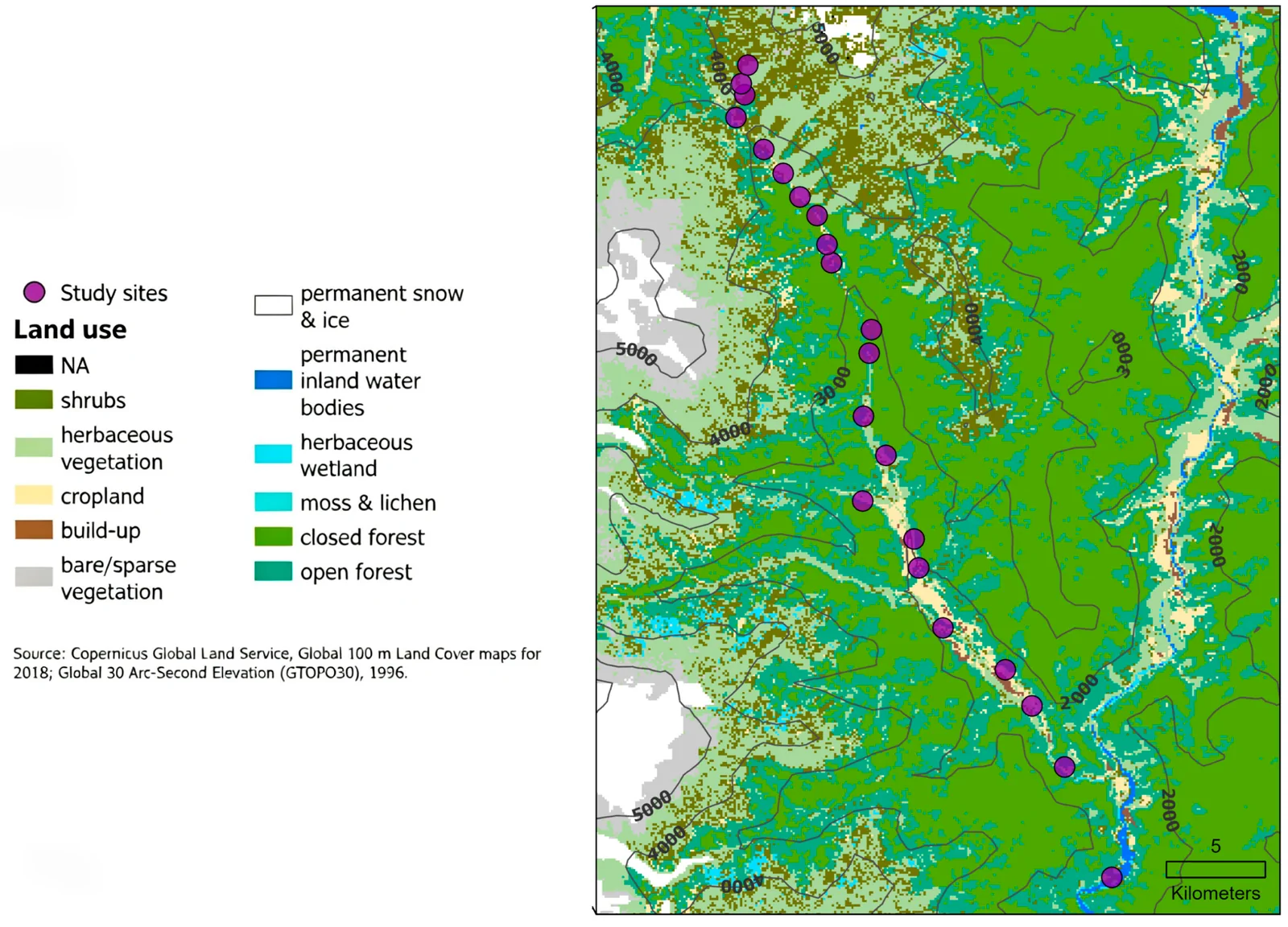
Map of the butterfly survey site in eastern range of Mount Gongga. The purple dots represent the positions of transects.

We established a total of 18 transects (Figure 2) along the highway connecting Moxi Town and Kangding City, extending northward to Yajiageng Ridge. The total survey route is approximately 50 km long, with elevations ranging from 1100 m to 4300 m. A sampling transect was arranged at every 200 m elevational interval. Each transect measured 5 m × 1000 m, spanning elevations from 1100 m to 4500 m.

We used a transect counting method commonly applied in European Butterfly Monitoring Schemes [42,43,44]. We recorded butterflies within 2.5 m to the left and to the right of the transect line (5 m width in total) while walking at steady pace of approximately 2.5 km/h during sunny conditions with a wind speed of 1 to 2 on the Beaufort scale. Each transect was visited in total 4 times from May to August 2012.

Species were either (a) identified by the wing or captured and identified in the field, or (b) sampled and identified in the lab. For all of the species identified, reference specimens were stored in the lab of the College of Life Sciences, China West Normal University. The identification of species was based on <Monographia Rhopalocerorum Sinensium> [45] and <Butterflies of China> [46]. For the transect data, see Table S1.

### 2.3. Environmental Data Sources

Climate data for this study were retrieved from WorldClim v2.1, while landscape data were extracted from EARTHDATA (https://search.earthdata.nasa.gov). All factors were extracted based on altitude. The scale of vegetation zone types was the same as that of altitude extraction method. For each elevation segment, the mean value of each factor was used. The data are provided in Table S2.

### 2.4. Data Analysis

Based on field survey data, five α-diversity indices were calculated, including species richness, abundance, Berger–Parker dominance index, Shannon–Wiener index, Simpson index, and Pielou evenness index [47,48]. Turnover, nestedness and the Simpson index were used to reflect β-diversity (reflects the degree of differences in species composition between different communities). Calculations were performed using the “vegan” and “betapart” packages in R 4.2.1.

Berger–Parker Dominance Index (Pi): The proportion of a specified species in a given habitat was calculated.

$$Pi=\frac{Ni}{N}\times 100\%$$

Ni: individual number of a specified species, and N: total individual number of all species. Dominant species indicator: Pi ≥ 5%, common species indicator: 5% > Pi ≥ 0.5%, and rare species indicator: Pi < 0.5%.

Shannon–Wiener Index (H): The structure of community diversity was described.

$$H=-\sum_{i=1}^{S}PilogPi$$

S: total species number, and Pi: dominance index.

Simpson Index (GS): Sample richness and evenness were comprehensively considered.

$$GS=1-\sum_{i=1}^{s}Pi^{2}$$

S: total species number, and Pi: dominance index.

Pielou Evenness Index (J): The distribution status of species in a community was measured.

$$J=\frac{H}{lnS}$$

H: Shannon–Wiener index, and S: total species number.

The beta diversity index (βSOR) is an index used to quantify beta diversity in ecological communities, reflecting the degree of differences in species composition between different communities. It can be decomposed into two components: turnover (βSIM) and nestedness (βSNE). The species turnover represents the substitution of species between different locations, where a species from one location is replaced by a different species from another location; nesting components represent the loss or acquisition of species between different locations, where the species composition of one location is a subset of the species composition of another location.

$$\beta SOR=\frac{b+c}{2a+b+c}$$

$$\beta SIM=\frac{min(b,c)}{a+min(b,c)}$$

$$\beta SNE=\frac{\left|b-c\right|}{2a+b+c}\times \frac{a}{a+min(b,c)}$$

a: number of species shared between two gradient zones A and B, b: number of species unique to gradient zone A, and c: number of species unique to gradient zone B.

### 2.5. Software

Data analysis was conducted in R 4.2.1. Species accumulation curves were plotted using the “specaccum” function in the “vegan” package. ANOVA was used to analyze the effects of altitude and vegetation zones on butterfly community richness and abundance, with linear fitting (lm) being performed when differences were significant. Extrapolation rarefaction analysis was conducted using the “iNEXT” package (q = 0 for richness, q = 1 for Shannon index, q = 2 for Simpson index) [49].

Bray–Curtis dissimilarity was calculated using the “vegan” package to analyze community dissimilarity.

Environmental data were processed using ArcGIS 10.2 for correlation analysis. Mantel tests were used to explore the relationships of climate, landscape, geographic distance, and combined environmental factors with species diversity. To precisely quantify the contributions of each factor to community similarity, geographic distance was converted using latitude and longitude and ln (x + 1) transformation, while environmental variables were calculated using Euclidean distance. Bray–Curtis distance, Euclidean distance, and Mantel tests were performed using the “vegan” package, geographic distance was calculated using the “distm” function in the “geosphere” package, and MRM analysis was performed using the “MRM” function in the “ecodist” package.

Faunal nomenclature follows the *Fauna Sinica* [50].

## 3. Results

### 3.1. Community Composition

A total of 864 butterflies representing 138 species belonging to five families and 80 genera were recorded. For the assemblage composition and biodiversity indices, see Table 1.

Table 1 shows that Nymphalidae had the highest Shannon–Wiener, Simpson and Pielou indices, indicating high α-diversity. Nymphalidae also had the highest Chao1 and ACE indices, indicating significantly higher butterfly abundance compared to other families. Papilionidae had the lowest indices, suggesting low α-diversity, with the lowest Pielou index indicating low evenness and significant relative abundance differences, with some species dominating.

### 3.2. Species Diversity Patterns of Butterfly Assemblage Composition Along Elevational Gradient

Species richness and abundance were found to decrease with increasing elevation. We found the highest species richness in the 1100–1500 m altitude range (71), followed by 1500–1900 m (66), and the lowest at 3500–3900 m and 3900–4300 m (4 each). In terms of abundance, 1100–1500 m had the highest (289 individuals) (EBF), followed by 1500–1900 m (240 individuals) (MEDBF), and the lowest at 4300–4500 m (9 individuals) (AMS), with 3500–3900 m (DCF to AMS) and 3900–4300 m (AM) having the same (10 individuals). 

**Figure 3:**
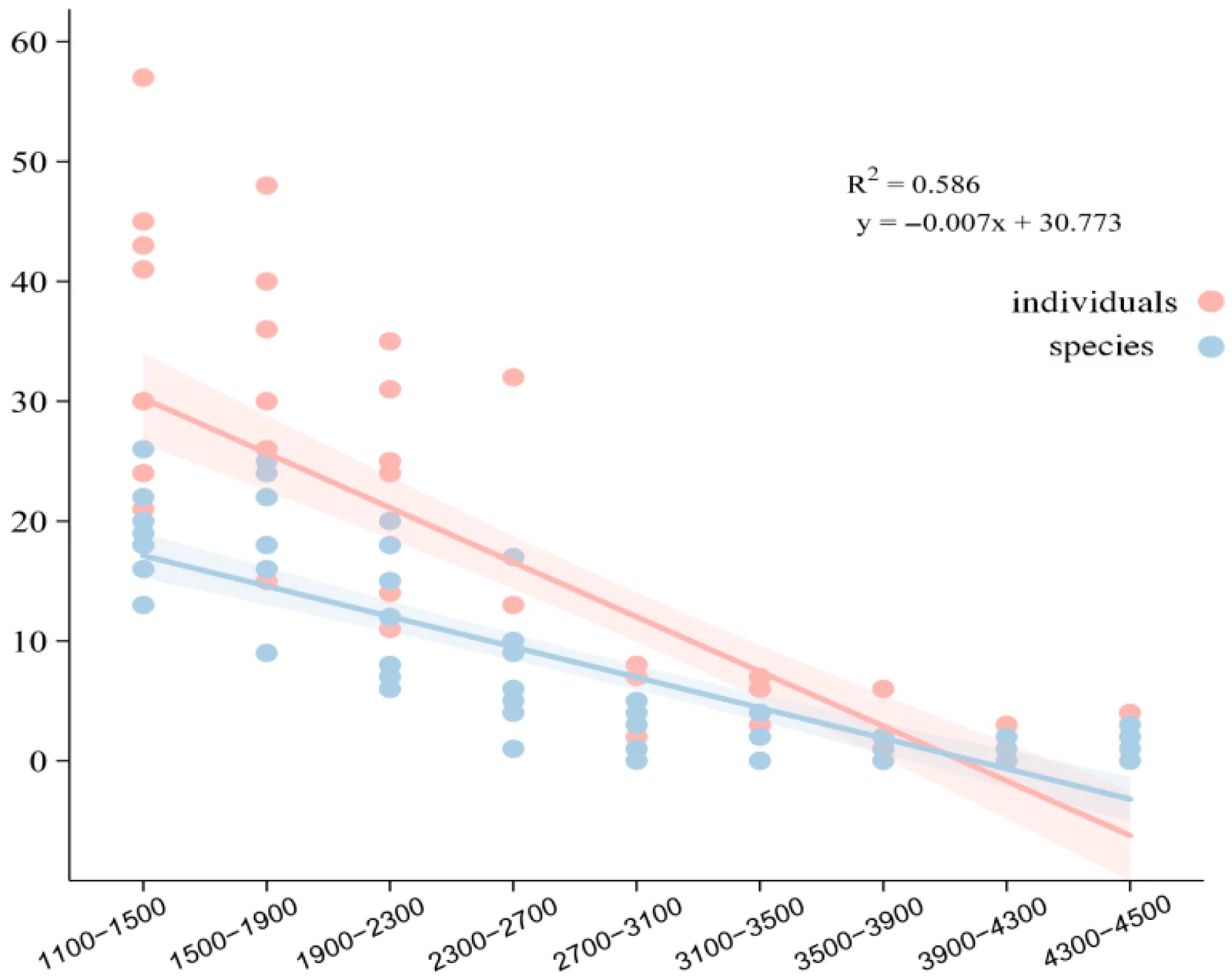
Linear fitting of abundance, species number and altitude.

Linear regression showed a significant negative correlation between species number and abundance with increasing elevation (coefficient: −0.007, *p* < 0.05) Figure 3.

**Figure 4:**
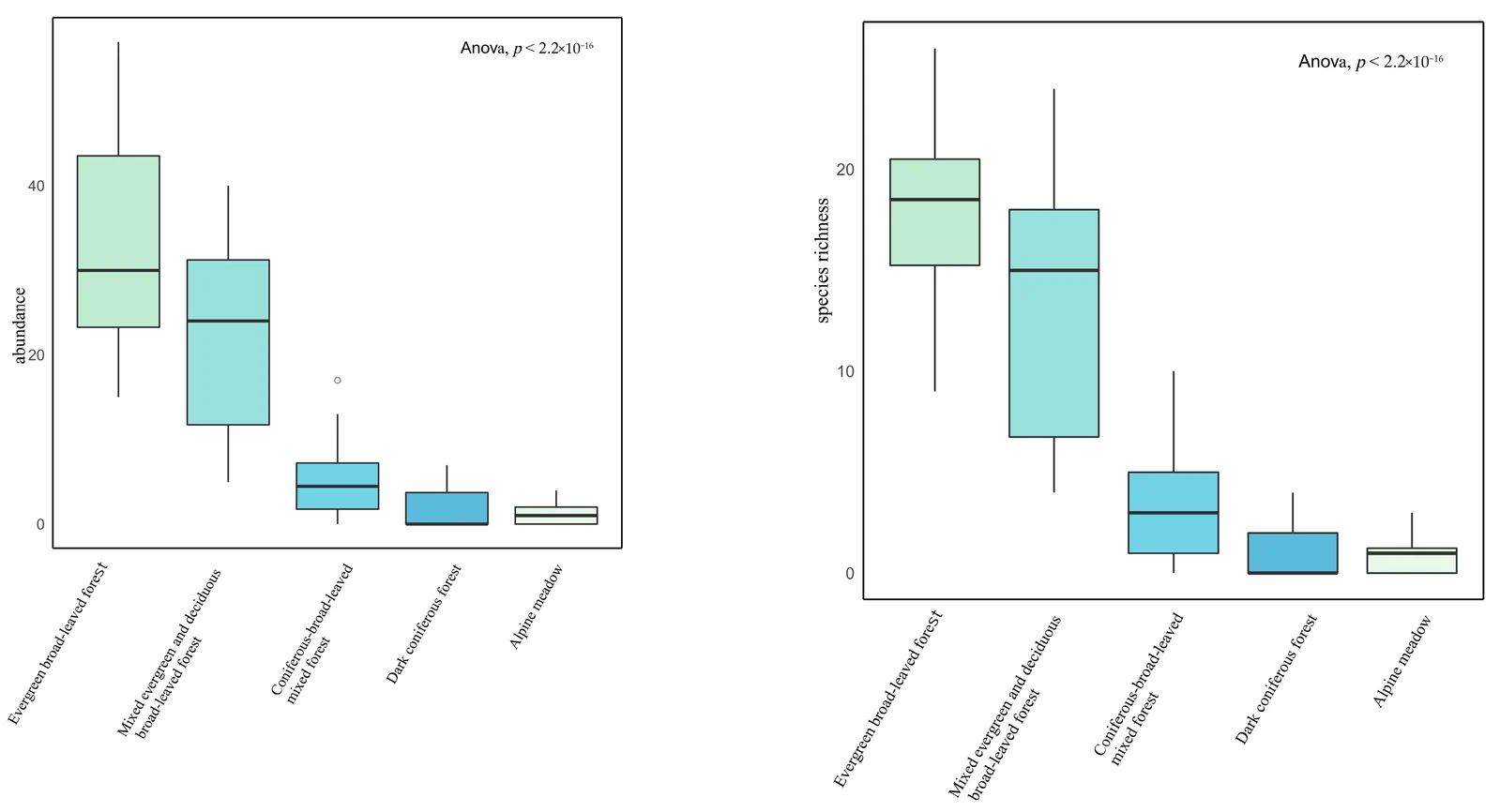
Intra-group differences in species abundance and species number at different elevations.

ANOVA showed significant differences in species richness and abundance across altitude ranges (*p* < 0.05) Figure 4.

### 3.3. α-Diversity Analysis Across Vegetation Zones

**Figure 5:**
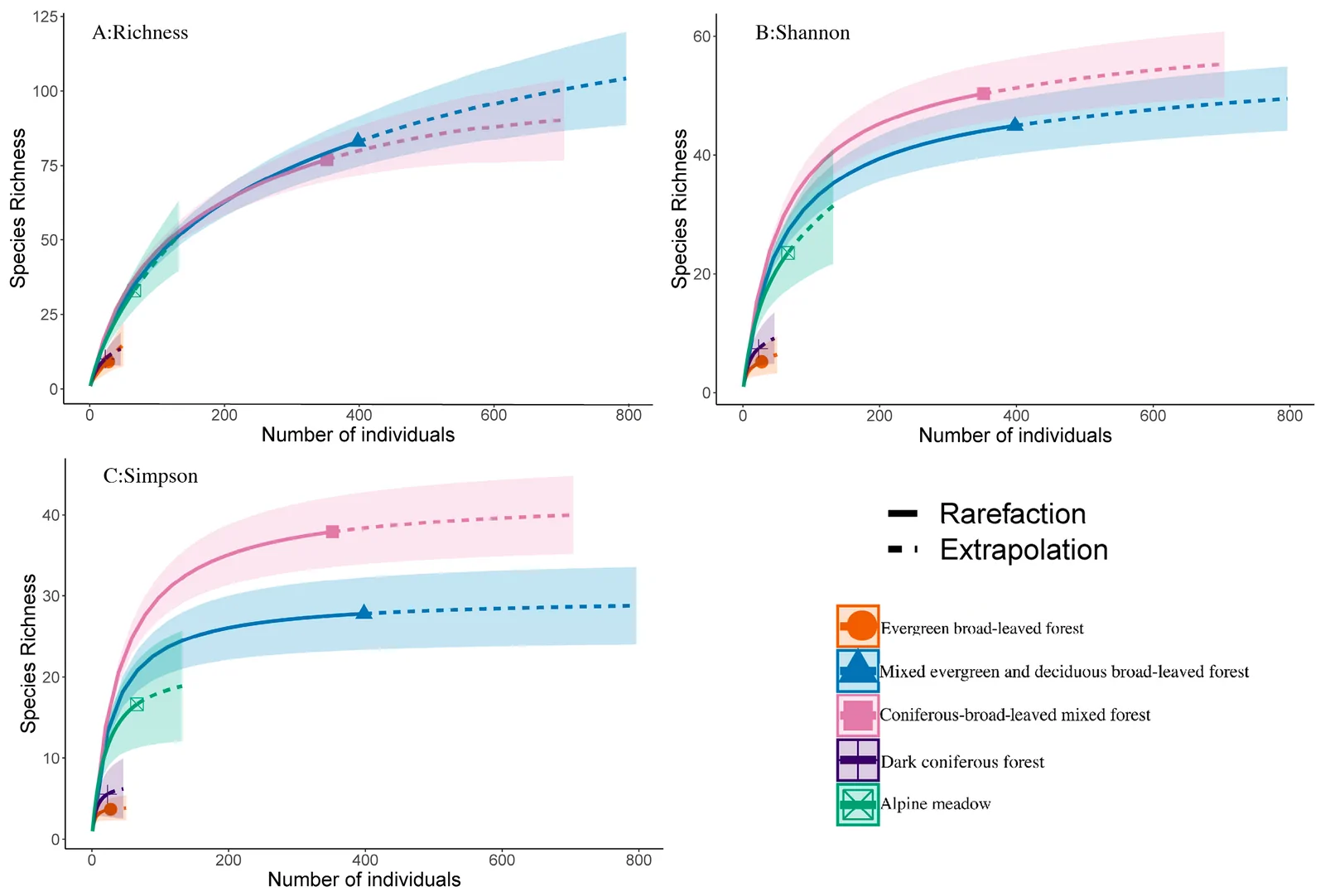
Sparse extrapolation of butterfly diversity in different vegetation zones. (**A**) Species richness; (**B**) Shannon index; (**C**) Simpson index.

Sparse extrapolation analysis revealed that significant differences in butterfly community diversity were found across vegetation zones. Although the EBF had the highest number of species, its extrapolated Shannon, Simpson, and richness indices were the lowest. The richness index was highest in the MEDBF, followed by the CBMF. The Shannon and Simpson indices followed the same order, CBMF > MEDBF > ASM > CF > EBF, as shown in Figure 5.

**Figure 6:**
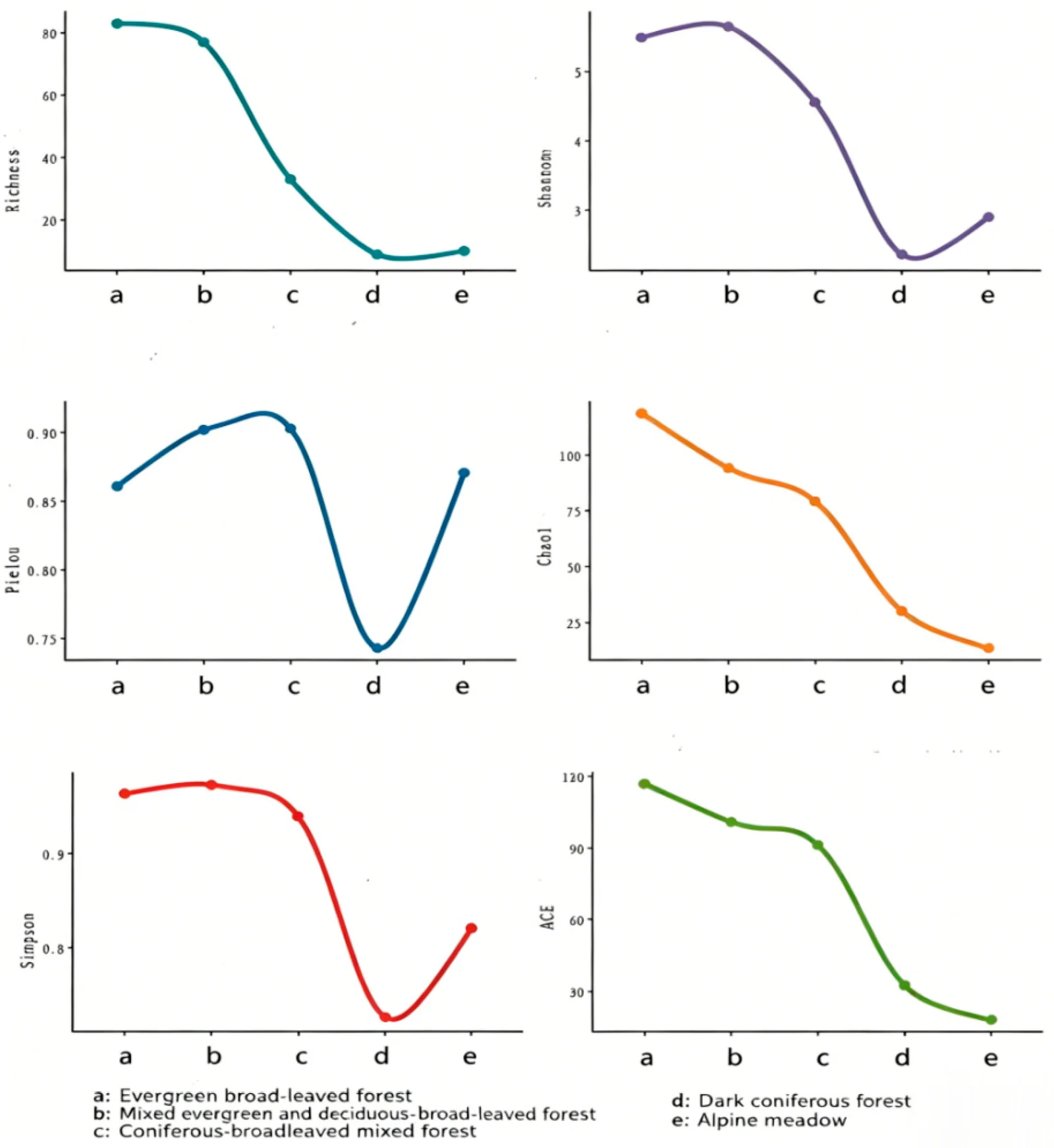
α-diversity of butterfly communities in different vegetation zones.

Diversity analysis indicated that the EDBMF had the highest Shannon and Simpson indices, followed by the Pielou index, indicating optimal α-diversity. The DCF had the lowest indices, suggesting the poorest α-diversity. The EBF had the highest Chao1 and ACE indices, indicating the greatest species abundance, while the AMS had the lowest. The richness, Shannon, Simpson, and Pielou indices showed an N-shaped pattern, reflecting an initial increase and subsequent decrease in α-diversity, with a rebound at the AMS. The Chao1 and ACE indices showed a linear decline with increasing elevation indicating a reduction in species richness with increasing altitude (Figure 6).

**Figure 7:**
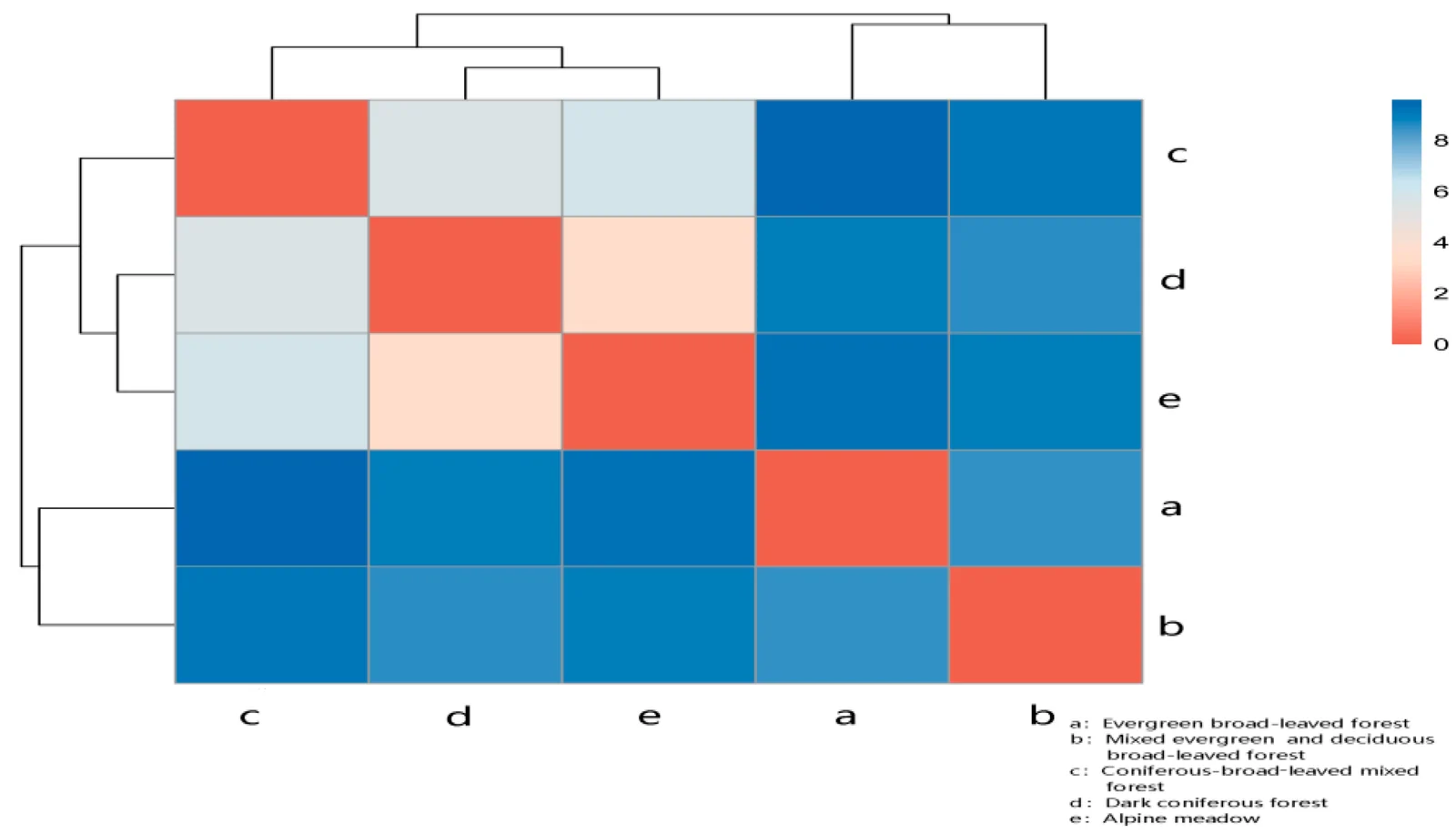
Similarity clustering of butterfly communities in different vegetation zones.

Correlation analysis showed that the dissimilarity coefficient between vegetation zones was generally >8, indicating that low similarity in butterfly communities was found. The EBF and MEDBF had a dissimilarity coefficient of 8.485, which was classified as similar. The DCF and AM had the highest similarity, with a dissimilarity coefficient of 3.606, and were grouped together (Figure 7).

### 3.4. β-Diversity

**Figure 8:**
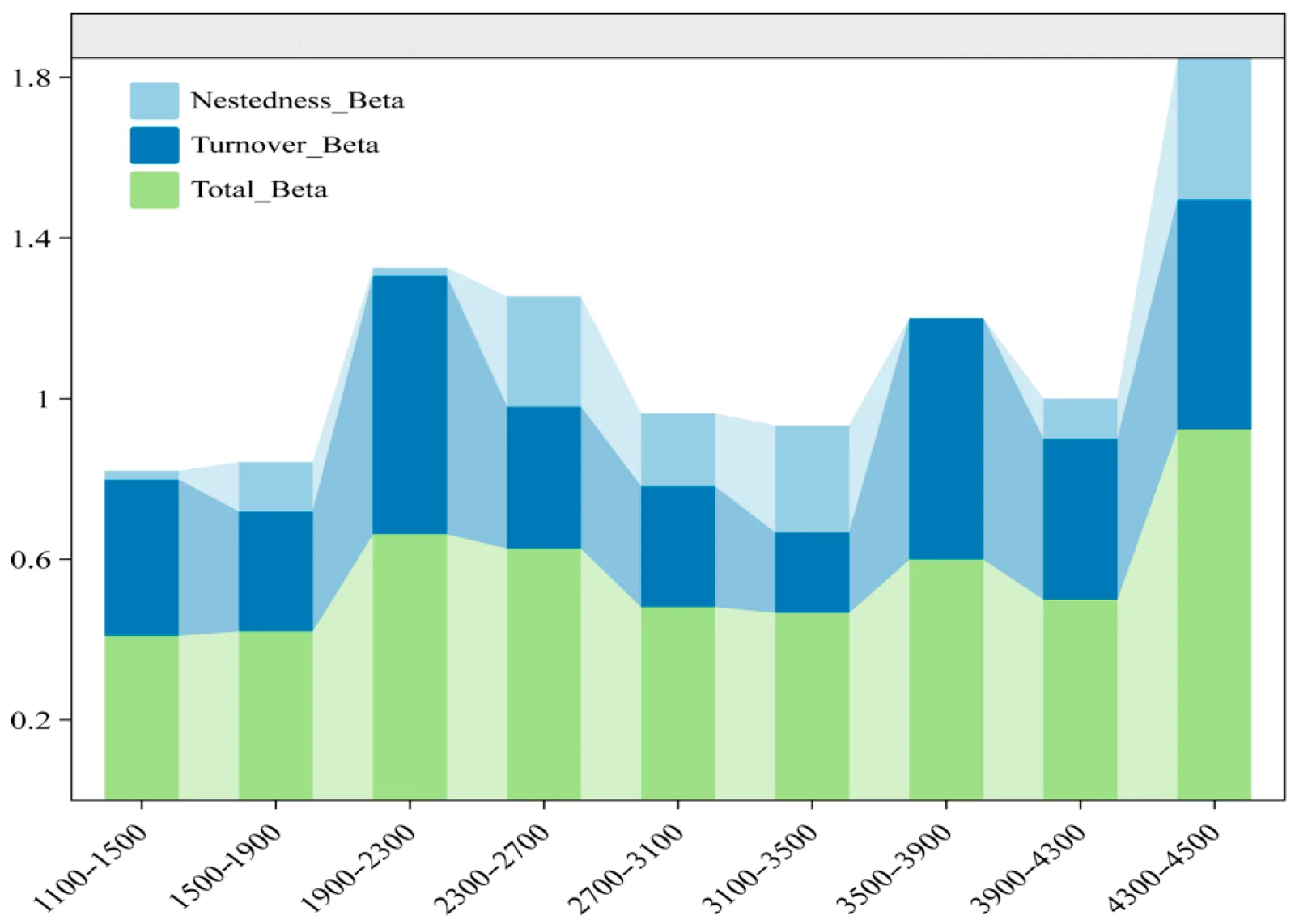
Changes in β-diversity and its components of butterfly community at different altitudes.

β-diversity analysis of butterfly assemblages on Mount Gongga revealed that low β-diversity was found, which was primarily driven by species turnover (βSIM), with significant species replacement being observed. The β-diversity (βSOR) along altitude was found to be nonlinearly distributed, with peaks at 1900–2300 m, 3500–3900 m, and 4300–4500 m. Species turnover (βSIM) followed a nonlinear distribution, with peaks at the same altitudes. Nestedness (βSNE) was nonlinear, with the highest peak being found at 2300–2700 m and decreasing to 0 at 3500–3900 m (Figure 8).

### 3.5. Driving Factors of Butterfly Diversity Patterns Along Elevational Gradients

**Figure 9:**
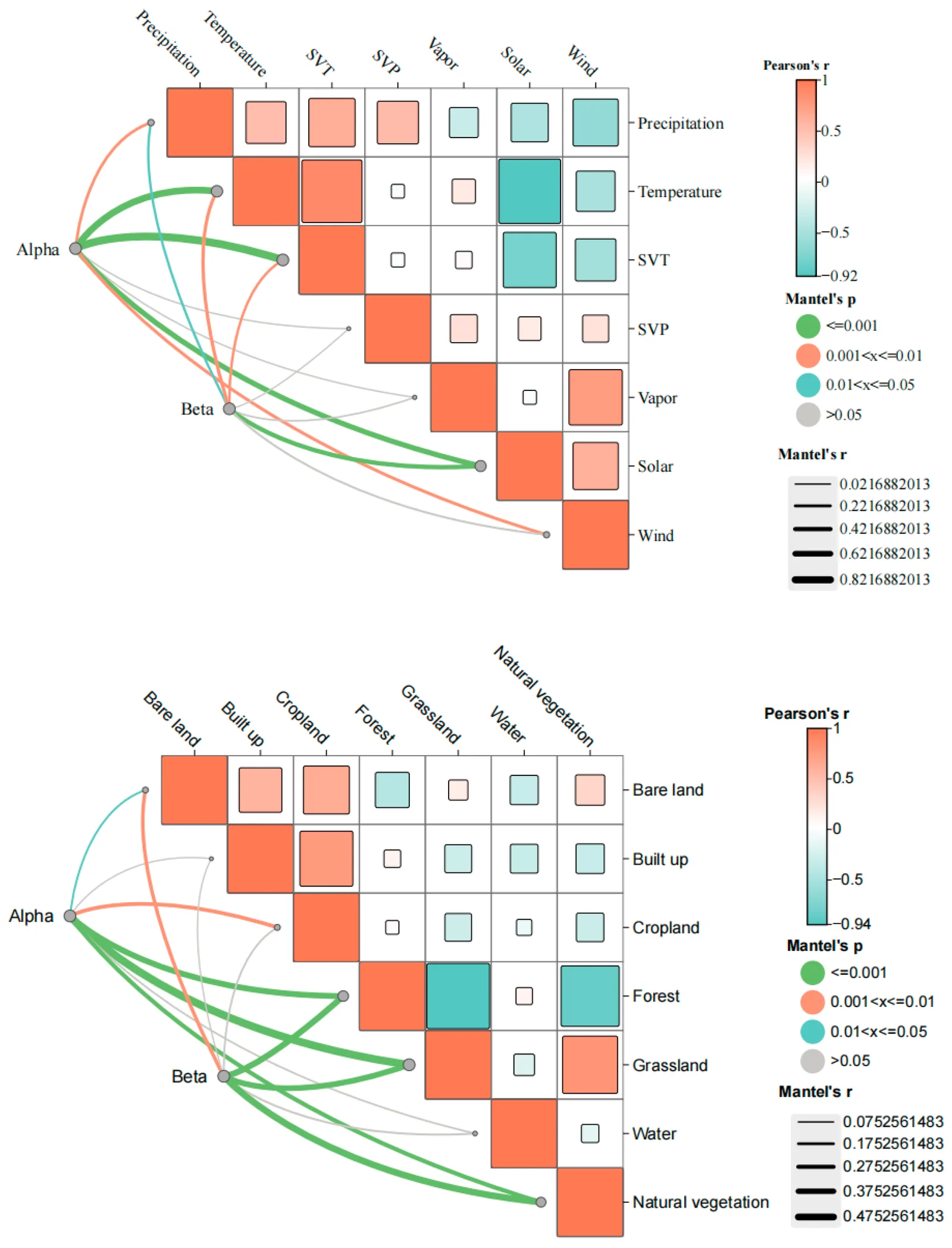
Correlation between predictor variables and α- and β-diversity indices at altitude scale.

Correlation analysis showed that butterfly community α- and β-diversity were significantly correlated with climatic, landscape, and spatial variables (*p* < 0.01). α-diversity was significantly correlated with annual precipitation, temperature, solar radiation, wind speed, and other environmental factors (*p* < 0.05), and highly significantly correlated with landscape factors such as farmland, forest, and grassland (*p* < 0.01), and water resource index (*p* < 0.05). β-diversity was highly significantly correlated with annual precipitation, monthly mean temperature, and solar radiation (*p* < 0.01), and significantly correlated with six landscape indices excluding farmland (*p* < 0.05) (Table 2 and Figure 9).

### 3.6. Drivers of Vertical Distribution Patterns of Butterflies Along Vegetation Zones

**Figure 10:**
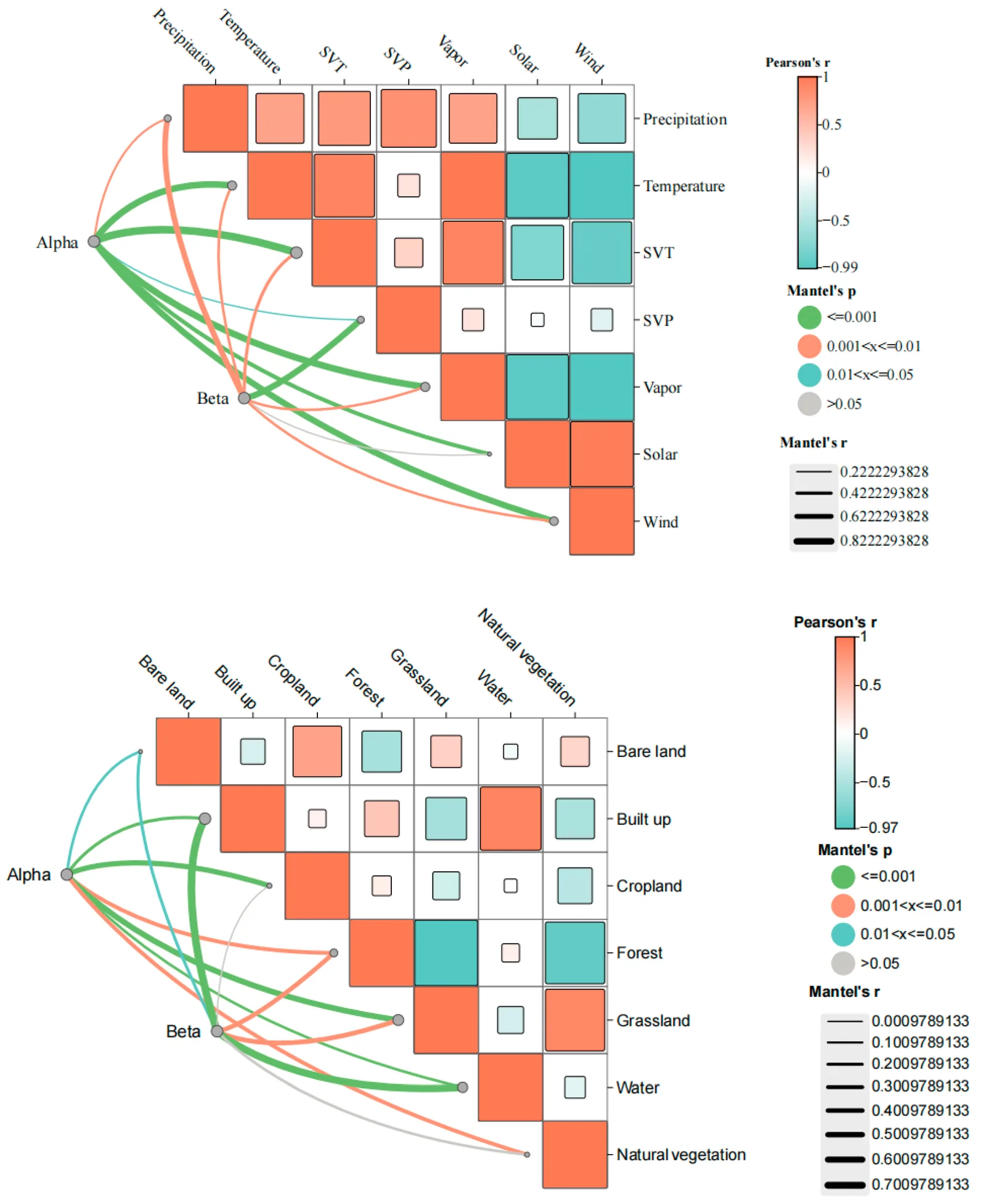
Correlation between predictor variables and α- and β-diversity indices at vegetation zone scale.

Correlation analyses revealed that the α-diversity of butterfly communities along vegetation zones exhibited highly significant correlations with composite environmental dissimilarity, landscape dissimilarity, and spatial distance (*p* < 0.001) (Table 3 and Figure 10).

In terms of environmental variables, the α-diversity of butterfly species showed highly significant correlations with annual precipitation, monthly mean temperature, seasonal temperature variation, monthly mean vapor pressure, monthly mean solar radiation, and monthly mean wind speed variation (*p* < 0.01) (Table 3, Figure 4 and Figure 5A). For landscape dissimilarity, variations in the cover indices of bare land, artificial construction, farmland, forest, grassland and natural vegetation were significantly correlated with butterfly α-diversity (*p* < 0.05) (Table 3 and Figure 10).

The species β-diversity (βSOR) of butterflies along vegetation zones was highly significantly correlated with composite environmental dissimilarity, landscape dissimilarity, and spatial distance (*p* < 0.001) (Table 3 and Figure 10). At the environmental level, species β-diversity (βSOR) had highly significant correlations with dissimilarities of all predictor variables (*p* < 0.01) (Table 3 and Figure 10). In terms of landscape dissimilarity, variations in the indices of bare land, artificial construction, forest and grassland all showed highly significant correlations with β-diversity (βSOR) (*p* < 0.01) (Table 3 and Figure 10).

**Figure 11:**
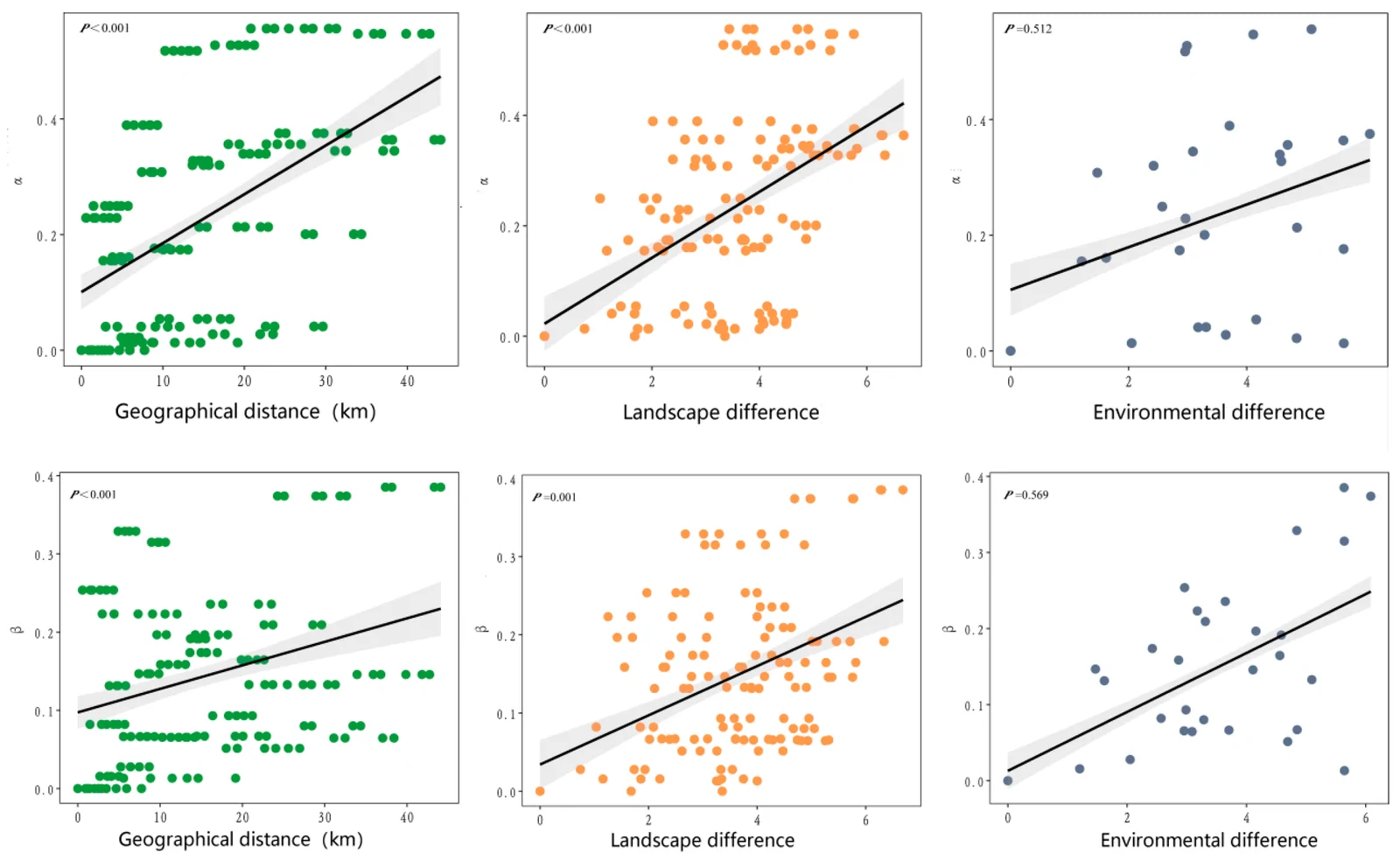
Distance attenuation effect between predictor variables and α- and β-diversity indices at altitude scale.

**Figure 12:**
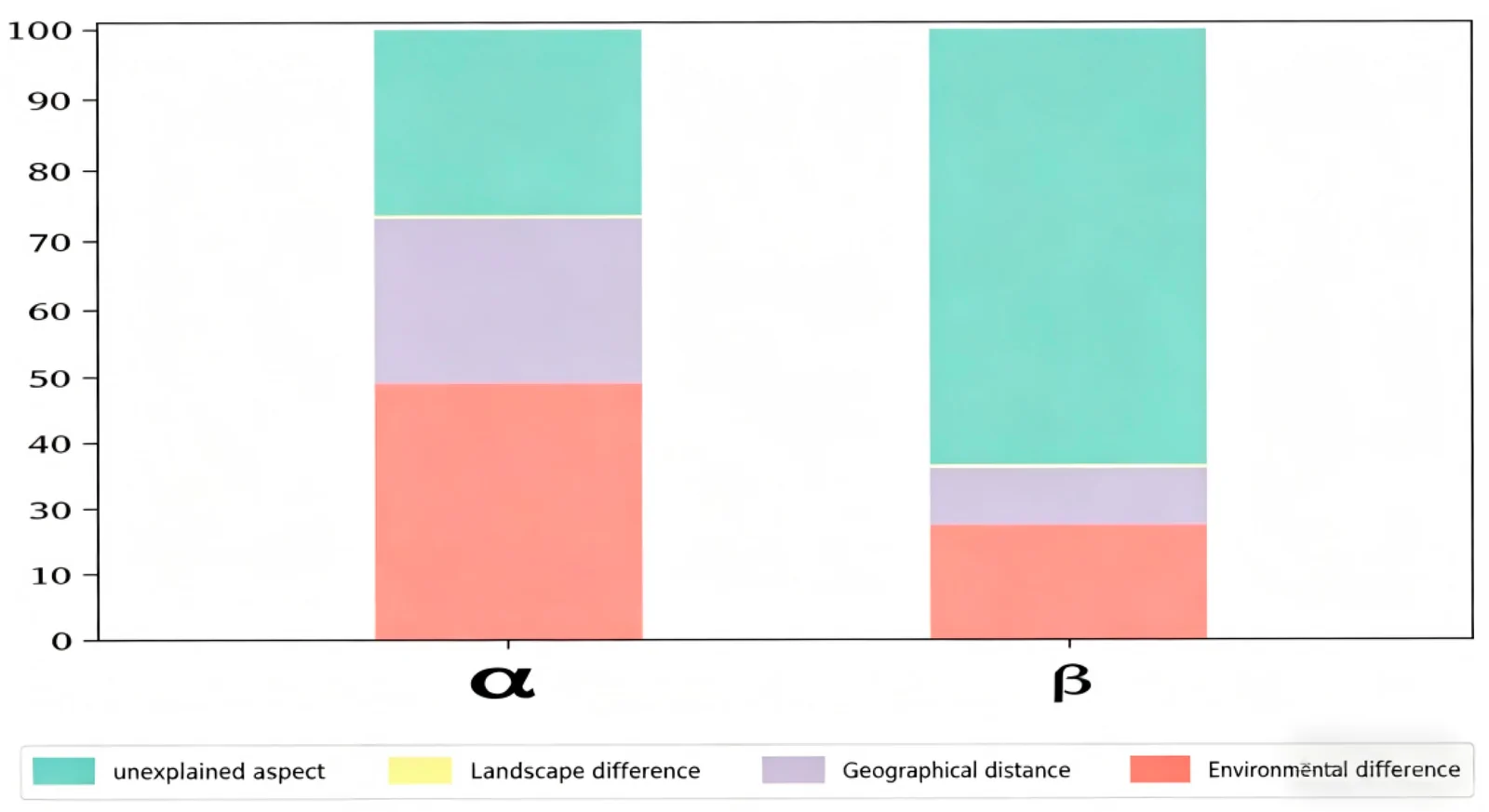
Interpretability of predictor variables and α- and β-diversity indices on the altitude scale.

MRM analysis indicated that environmental differences and geographic distance were the primary driving factors for the elevational alpha diversity patterns of butterfly assemblages on Gongga Mountain, with no distance decay effect being observed. Environmental differences had the highest contribution (44%), followed by geographic distance (27%), and landscape differences had the least impact (0.5%). Environmental differences and geographic distance were found to significantly influence the changes in butterfly β-diversity along the elevational gradient, with no distance decay effect being observed. Environmental differences had the highest contribution (19%), followed by geographic distance (10%), and landscape differences had the least impact (0.5%) (Figure 11 and Figure 12).

## 4. Discussion

### 4.1. Butterfly Diversity Characteristics of Mount Gongga

A total of 138 butterfly species belonging to 80 genera and five families were recorded on Mount Gongga in this study, accounting for 23% of the more than 600 known butterfly species in the Hengduan Mountains region [51]. Nymphalidae was the dominant family in the local butterfly community, which is consistent with most previous studies on butterfly diversity.

### 4.2. Elevational Patterns of Alpha and Beta Diversity of Butterfly Communities

Based on standardized transect surveys across Mount Gongga, this study systematically explored the elevational distribution patterns of butterfly alpha and beta diversity along vegetation zonation gradients from two dimensions of elevation and vegetation distribution. The results showed that both species richness and abundance of butterfly communities decreased significantly with increasing elevation, which supports the classical conclusion that insect diversity declines with elevation in most mountain systems [10,16,52]. This is consistent with our hypothesis (1). In contrast, butterfly beta diversity exhibited a unique nonlinear trimodal distribution along the elevational gradient, with distinct peaks at 1900–2300 m, 3500–3900 m, and 4300–4500 m. This pattern differs from the monotonically decreasing or unimodal beta diversity trends reported for most mountain insect communities. The observed pattern is inconsistent with our prior hypothesis (1). (2) Elevationally stratified vegetation belts shape differentiated host plant assemblages, driving distinct butterfly community differentiation and intense species turnover across elevations. (3) High species turnover of butterfly communities is one of the dominant factors shaping the beta diversity pattern of Mount Gongga.

### 4.3. Formation Mechanisms of Vertical Distribution Patterns of Butterfly Alpha and Beta Diversity

The unique elevational distribution patterns of alpha and beta diversity of butterfly communities on Mount Gongga are synergistically shaped by vertical hydrothermal differentiation, vegetation ecotone effects, habitat heterogeneity, interspecific ecological adaptation, and high species turnover rates [1,6].

#### 4.3.1. Mechanism Underlying the Elevational Pattern of Alpha Diversity

The elevational decline in butterfly alpha diversity on Mount Gongga is mainly regulated by environmental filtering, resource availability, and differential species adaptation [5,10,52]. High-altitude habitats are characterized by low temperatures, strong winds, and short growing seasons, forming harsh and unstable alpine climatic conditions. Such rigorous environmental filtering severely restricts the survival and reproduction of thermophilic and generalist species, retaining only cold-tolerant alpine specialists such as Parnassiidae, thereby significantly reducing butterfly species richness and abundance at high elevations [27,52]. Meanwhile, the continuous decline in vegetation diversity, coverage, and structural complexity with elevation causes severe shortages of larval host plants and adult nectar resources, reducing habitat carrying capacity and limiting multi-species coexistence, which serves as the core driver of alpha diversity loss at high elevations [39]. In comparison, low- and mid-altitude regions possess favorable hydrothermal conditions and high habitat heterogeneity, supporting diverse butterfly species with different feeding habits and habitat preferences. In contrast, high-altitude habitats with simple structures and limited resources only accommodate a small number of specialized species with narrow ecological niches. Collectively, climatic filtering, resource scarcity, and interspecific adaptive differences shape the consistent declining elevational pattern of butterfly alpha diversity on Mount Gongga.

#### 4.3.2. Mechanism Underlying the Elevational Pattern of Beta Diversity

Unlike the monotonically decreasing trend of alpha diversity, butterfly beta diversity on Mount Gongga presents a distinctive trimodal distribution pattern, primarily driven by vegetation ecotone effects, habitat heterogeneity differentiation, elevational species turnover, and unique topographic microclimates of the Hengduan Mountains [6]. The first beta diversity peak occurs at 1900–2300 m, the ecotone between evergreen broad-leaved forests and mixed coniferous and broad-leaved forests. This elevational zone features optimal hydrothermal conditions and complex vertical vegetation structures, supporting the coexistence and frequent replacement of low-altitude thermophilic species and mid-altitude generalist species, resulting in high species turnover and low community similarity. The second peak appears at 3500–3900 m, the transitional zone from dark coniferous forests to alpine shrubs. The gradual transformation from closed-canopy forest habitats to open alpine shrublands leads to the decline of forest-dependent butterflies and the replacement by open-habitat alpine species, and the abrupt habitat shift triggers rapid community reorganization. The third peak is located at 4300–4500 m, the ecotone between alpine meadows and bare rocky habitats. This extreme high-altitude zone exclusively supports highly cold-tolerant specialist butterflies (e.g., Parnassiidae) with the complete disappearance of low- and mid-elevation species. The extremely high habitat specificity and unique community composition maximize inter-elevational dissimilarity, forming the third beta diversity peak. This is also consistent with hypothesis (2). Furthermore, the complex terrain and fragmented microclimates of the central Hengduan Mountains alleviate environmental homogenization at mid- and high elevations, further accelerating species turnover and ultimately forming the unique trimodal beta diversity pattern.

### 4.4. Drivers of High Species Turnover of Butterfly Communities

Butterfly communities on Mount Gongga exhibit extremely high species turnover rates along the elevational gradient, which fundamentally accounts for the multimodal pattern of beta diversity. This prominent turnover pattern is comprehensively driven by abrupt habitat transitions, high microclimatic heterogeneity, intense niche differentiation, and complex topographic barriers in the Hengduan Mountains [1,6,53,54,55]. Mount Gongga has a complete vertical vegetation spectrum, where rapid habitat alternation within narrow elevational ranges prevents community homogenization and promotes continuous species replacement [6,55]. First, ecotone interface effects accelerate community turnover. Alternating vegetation zones of forests, shrubs, meadows, and bare rocks lead to abrupt changes in host plant assemblages, eliminating habitat-specialized species and facilitating the colonization of alpine-adapted species, thus enhancing species replacement intensity [6]. Second, complex topography generates highly heterogeneous microhabitats. Deep valleys, variable slope aspects, and water vapor convergence create fragmented local microclimates. Significant differences in temperature, humidity, and light within the same elevational belt prevent habitat homogenization and sustain persistent community differentiation among adjacent elevational zones [56]. Third, butterfly species exhibit strong vertical niche stratification, consisting of low-altitude thermophilic species, mid-altitude widespread species, and high-altitude cold-tolerant specialists. These functional groups differ substantially in thermal tolerance, host preference, and habitat adaptation, such that minor elevational fluctuations can trigger substantial community restructuring. Fourth, stratified environmental filtering maintains continuous species replacement. Environmental stress varies distinctly across elevations: low and mid-elevations are mainly filtered by resource competition and vegetation structure, whereas high elevations are dominated by extreme climatic filtering such as low temperature and strong wind. Such stratified filtering results in completely different dominant species among elevational belts and sustains persistent community turnover along the entire gradient [18]. Fifth, Nymphalidae butterfly was the dominant family in the local butterfly community, which is consistent with most previous studies on butterfly diversity. Owing to their strong dispersal capacity and wide environmental adaptability, Nymphalidae is one of the most widely distributed butterfly groups in global mountain ecosystems, which explains one of the reason for high beta diversity of the butterfly community of Mount Gongga. This is also consistent with hypothesis (3).

### 4.5. Conservation Implications and Suggestions

#### 4.5.1. Zoned and Differentiated Conservation Focusing on Core Vegetation Transition Zones

Consistent with the trimodal beta diversity pattern identified in this study, the three vegetation transition zones of 1900–2300 m, 3500–3900 m, and 4300–4500 m represent critical hotspots for butterfly species turnover and community differentiation, characterized by rapid species replacement and high community uniqueness, and thus constitute priority conservation areas. For the low-altitude mixed forest transition zone (1900–2300 m), human disturbances including understory exploitation and tourist trampling should be strictly regulated to protect complex forest structures and diverse microhabitats and maintain the integrity of butterfly host and nectar plant communities. For the mid–high-altitude forest–shrub ecotone (3500–3900 m), overgrazing should be prohibited to conserve forest–shrub habitat interfaces and sustain the successional alternation between forest-dwelling and alpine butterfly communities. For the alpine meadow–rock transition zone (4300–4500 m), excessive grazing and anthropogenic activities should be restricted to protect the habitats of endemic and rare alpine butterflies such as Parnassiidae and prevent species extinction caused by extreme habitat degradation. Elevationally zoned and precise management enables differentiated conservation of heterogeneous butterfly communities and effectively safeguards core butterfly biodiversity hotspots in the region.

#### 4.5.2. Maintaining Vegetation Integrity to Sustain Butterfly Resource Supply

Vegetation diversity and the abundance of host and nectar plants are core determinants of the elevational pattern of butterfly alpha diversity and serve as the fundamental basis for stable butterfly communities. In low- and mid-altitude areas, native broad-leaved and mixed forest communities should be strictly protected to avoid the replacement of indigenous vegetation by monocultural artificial plantations. Retaining understory shrubs and wild herbs is essential to enrich food resources for butterfly larvae and adults. In high-altitude regions, priority should be given to the conservation of native alpine meadows and shrubs to prevent grassland degradation and bare land expansion and maintain the resource carrying capacity of fragile high-altitude habitats. Sustaining regional vegetation integrity can provide stable food and habitat resources for butterfly communities across elevations and ensure long-term community stability.

#### 4.5.3. Addressing Climate Change to Enhance Conservation of High-Altitude Endemic Species

Long-term constraints of extreme alpine environments, including low temperatures and short growing seasons, have fostered numerous cold-specialized butterfly species with narrow ecological niches and high sensitivity to climate change. Climate warming induces upward habitat contraction and area loss for high-altitude butterflies, potentially leading to population decline and local extinction of endemic species [11,13]. For the endemic butterfly communities at 4300–4500 m on Mount Gongga, a long-term dynamic ecological monitoring system should be established to track continuous changes in regional climate, vegetation cover, and butterfly community structure. Targeted conservation research should be conducted for rare and endangered alpine butterflies (e.g., Parnassiinae), and a regional species resource database should be constructed. Artificial habitat restoration and auxiliary conservation measures can be implemented for vulnerable populations to improve the resistance and ecological stability of high-altitude butterfly communities.

#### 4.5.4. Establishing Long-Term Monitoring Mechanisms to Improve Biodiversity Conservation Systems

Species turnover and community differentiation of mountain butterflies are dynamic ecological processes, and long-term fixed monitoring is essential for clarifying community succession and optimizing conservation strategies. Based on the elevational distribution patterns of butterflies on Mount Gongga, permanent monitoring transects should be established across the trimodal distribution along the elevational gradient, with distinct peaks at 1900–2300 m, 3500–3900 m, and 4300–4500 m typical vegetation belts to regularly investigate butterfly species composition, population abundance, vegetation status, and habitat environmental factors, thereby systematically revealing the spatiotemporal dynamics of butterfly communities. Combined with regional ecological conservation policies, multiple measures including forest protection, grassland restoration, and rare species conservation should be integrated to construct a comprehensive conservation system featuring “zoned protection, targeted restoration, dynamic monitoring, and long-term management”, so as to realize the sustainable conservation and development of butterfly resources and mountain ecosystems on Mount Gongga [44].

## Figures and Tables

**Table 1:** α-diversity indices of butterflies in different families on Mount Gongga.

Family	Richness	Shannon	Simpson	Pielou	Chao1	ACE
Papilionidae	12	2.66	0.76	0.74	15.00	13.98
Pieridae	17	3.20	0.87	0.78	32.00	23.93
Nymphalidae	65	5.20	0.96	0.86	94.25	99.41
Lycaenidae	28	3.88	0.90	0.81	30.14	32.19
Hesperiidae	16	3.35	0.87	0.84	20.20	27.31

**Table 2:** Correlations between predictor variables and alpha and beta diversity indices at the elevational-band scale.

Predictor Variables	α-Diversity	β-Diversity
Annual precipitation	0.443 ***	0.323 **
Monthly mean temperature	0.678 ***	0.329 ***
Temperature seasonality (SVT)	0.933 ***	0.098
Precipitation seasonality (SVP)	−0.012	0.079
Monthly mean vapor pressure	−0.027	−0.046
Monthly mean solar radiation	0.662 ***	0.560 ***
Monthly mean wind speed	0.167 *	0.079
Bare land	0.126	0.465 ***
Built-up land	0.123	0.329 **
Cropland	0.289 **	0.110
Forest	0.530 ***	0.448 ***
Grassland	0.630 ***	0.433 ***
Water	0.173 *	0.258 *
Natural vegetation	0.667 ***	0.470 ***
Geographic distance	0.586 ***	0.305 **
Landscape dissimilarity	0.353 ***	0.490 ***
Combined environmental factors	0.499 ***	0.341 ***

Note: * *p* < 0.05; ** *p* < 0.01; *** *p* < 0.001.

**Table 3:** Correlation between predictor variables and α- and β-diversity indices at vegetation zone scale.

Predictor Variables	α-Diversity	β-Diversity
Precipitation	0.436 **	0.832 ***
Temperature	0.756 ***	0.459 **
Temperature seasonality (SVT)	0.878 ***	0.443 ***
Precipitation seasonality (SVP)	0.161	0.779 ***
Monthly mean of vapor	0.786 ***	0.420 **
Monthly mean of solar radiation	0.634 ***	0.527 **
Monthly mean of wind speed	0.733 ***	0.512 **
Bare land	0.305 **	0.332 **
Built-up	0.191 *	0.996 ***
Cropland	0.530 ***	0.155
Forest	0.293 **	0.535 ***
Grassland	0.527 ***	0.461 ***
Water	0.140	0.929
Natural vegetation	0.619 ***	0.375
Geographical distance	0.591 ***	0.638 ***
Landscape difference	0.355 **	0.766 ***
Combined environmental factors	0.565 ***	0.748 ***

Note: * *p* < 0.05; ** *p* < 0.01; *** *p* < 0.001.

## Data Availability

The original contributions presented in this study are included in the Supplementary Material. Further inquiries can be directed to the corresponding author.

## Supplementary Materials

The following supporting information can be downloaded at: https://www.mdpi.com/article/10.3390/insects17090890/s1, Table S1: Transect counting data; Table S2: Environment factors.
